# *BCL7A* is silenced by hypermethylation to promote acute myeloid leukemia

**DOI:** 10.1186/s40364-023-00472-x

**Published:** 2023-03-20

**Authors:** Juan Rodrigo Patiño-Mercau, Carlos Baliñas-Gavira, Alvaro Andrades, Maria S. Benitez-Cantos, Ana Ercegovič Rot, Maria Isabel Rodriguez, Juan Carlos Álvarez-Pérez, Marta Cuadros, Pedro P. Medina

**Affiliations:** 1 Gene Expression Regulation and Cancer Group (CTS-993), GENYO, Centre for Genomics and Oncological Research: Pfizer-University of Granada-Andalusian Regional Government, Granada, Spain; 2 Department of Biochemistry and Molecular Biology I, Facultad de Ciencias, University of Granada, Avda. de Fuentenueva S/N, 18071 Granada, Spain; 3 Present Address: Institut Curie, Paris Sciences Et Lettres Research University, Sorbonne University, INSERM U934/CNRS UMR3215, Paris, France; 4 Health Research Institute of Granada (Ibs.Granada), Granada, Spain; 5 Department of Biochemistry and Molecular Biology III and Immunology, University of Granada, Granada, Spain; 6 Present Address: International Postgraduate School Jožef Stefan, Ljubljana, Slovenia; 7 Department of Biochemistry and Molecular and Structural Biology, Jožef Stefan Institute, Ljubljana, Slovenia

**Keywords:** SWI/SNF complex, AML, Tumor suppressor, DNA hypermethylation

## Abstract

**Background:**

Recent massive sequencing studies have revealed that SWI/SNF complexes are among the most frequently altered functional entities in solid tumors. However, the role of SWI/SNF in acute myeloid leukemia is poorly understood. To date, SWI/SNF complexes are thought to be oncogenic in AML or, at least, necessary to support leukemogenesis. However, mutation patterns in SWI/SNF genes in AML are consistent with a tumor suppressor role. Here, we study the SWI/SNF subunit BCL7A, which has been found to be recurrently mutated in lymphomas, but whose role in acute myeloid malignancies is currently unknown.

**Methods:**

Data mining and bioinformatic approaches were used to study the mutational status of *BCL7A* and the correlation between BCL7A expression and promoter hypermethylation. Methylation-specific PCR, bisulfite sequencing, and 5-aza-2'-deoxycytidine treatment assays were used to determine if BCL7A expression was silenced due to promoter hypermethylation. Cell competition assays after BCL7A expression restoration were used to assess the role of BCL7A in AML cell line models. Differential expression analysis was performed to determine pathways and genes altered after BCL7A expression restoration. To establish the role of BCL7A in tumor development in vivo, tumor growth was compared between BCL7A-expressing and non-expressing mouse xenografts using in vivo fluorescence imaging*.*

**Results:**

BCL7A expression was inversely correlated with promoter methylation in three external cohorts: TCGA-LAML (*N* = 160), TARGET-AML (*N* = 188), and Glass et al. (2017) (*N* = 111). The AML-derived cell line NB4 silenced the BCL7A expression via promoter hypermethylation. Ectopic BCL7A expression in AML cells decreased their competitive ability compared to control cells. Additionally, restoration of BCL7A expression reduced tumor growth in an NB4 mouse xenograft model. Also, differential expression analysis found that *BCL7A* restoration altered cell cycle pathways and modified significantly the expression of genes like *HMGCS1, H1-0*, and *IRF7* which can help to explain its tumor suppressor role in AML.

**Conclusions:**

BCL7A expression is silenced in AML by promoter methylation. In addition, restoration of BCL7A expression exerts tumor suppressor activity in AML cell lines and xenograft models.

**Supplementary Information:**

The online version contains supplementary material available at 10.1186/s40364-023-00472-x.

## Background

Acute myeloid leukemia (AML) comprises a group of heterogeneous hematopoietic malignancies derived from myeloid precursors and associated with poor outcomes. Non-random cytogenetic aberrations are the single most important prognostic factor of AML [1]. The mutational burden of AML is among the lowest compared with other tumor types, which suggests that AML may have a high dependency on epigenetic mechanisms, such as aberrant methylation or alterations in chromatin remodeling [2].

The Switch/Sugar Non-Fermenting (SWI/SNF) complexes are chromatin-remodeling elements that use the energy released by ATP hydrolysis to alter the interactions between DNA and histones, modifying the accessibility of DNA information to the cell machinery and therefore playing a significant role in gene expression [3]. At least 29 different subunits have been identified in mammalian SWI/SNF complexes, and they can assemble in different combinations to create different functional complexes [4]. SWI/SNF complexes play important roles in cell identity and in cell differentiation, including a relevant role in hematological stem cell self-renewal and lineage differentiation [5, 6].

Massive sequencing studies have revealed that SWI/SNF complexes are among the most recurrently mutated functional elements across all tumor types, reaching an overall mutation rate of 25% [7]. Mutations found in cancer-related SWI/SNF subunits are mostly deleterious [8], consistent with the widespread tumor suppressor function of the complex. Accordingly, sequencing studies in AML have also found tumor suppressor-like mutation patterns in SWI/SNF genes [9, 10]. However, much of the experimental evidence developed in AML so far suggest that the SWI/SNF complex may be necessary to sustain leukemogenesis [11, 12].

The subunit BCL7A often undergoes functional inactivation in Diffuse Large B cell lymphoma (DLBCL) because of bi-allelic mutations that damage its tumor suppression abilities [3, 13]. However, we lack studies to determine the role of *BCL7A* in acute myeloid-derived neoplasias. Also, to understand the role of SWI/SNF complexes in AML, it is necessary to complement sequencing studies with functional studies.

In the present study, we show the first experimental evidence of a tumor suppressor role of BCL7A in vivo and in vitro in AML, finding that some tumors silence *BCL7A* expression by promoter methylation and presenting the first functional models that support its tumor suppressor role.

## Methods

### Cell culture

NB4 and M-07e cell lines were purchased from DSMZ. Cell lines were cultured at 37 °C with a 5% CO_2_ atmosphere. They were grown in RPMI 1640 medium (Cat#L0498-500, Biowest, Riverside, MO, USA) supplemented with 10% fetal bovine serum (Cat#10,270–106, Gibco™ Thermo Fisher Scientific), and 100 U/mL streptomycin and penicillin (Cat#P0781-100ML, Sigma-Aldrich). The NB4 cell line used for in vitro and in vivo experiments was subjected to mycoplasma contamination analysis using Venor GeM-qEP (Minerva Biolabs, Berlin, Germany).

### Methylation-specific PCR

We performed a methylation-specific PCR (MSP) with bisulfite converted DNA. We used two commercial genomic DNA that were completely methylated (Methylated gDNA) and completely unmethylated (Unmethylated gDNA) as controls. We used Kapa HiFi Hot start Uracil + Ready mix PCR kit (Kapa Biosystems). The primers obtained from Okada et al. [14] were: for the methylated sequence: MSP FW (M): 5´- GGTAGGCGACGTTTTAGTTC -3’ and MSP RV (M): 5’-GAATTAAAAACACCGATTCG -3’; for the unmethylated sequence: MSP FW (U): 5’-TGGGGTAGGTGATGTTTTAGTTT -3’ and MSP RV (U): 5’-CCAAATTAAAAACACCAATTCAA-3’. Their location relative to the *BCL7A* start codon was 688 bp upstream for the 3’ end nucleotide of the FW primers, and 598 bp upstream for the first G closest to the 5’ end of the RV primers. The location of the amplicon was: chr12:122,459,288–122,459,402, GRCh37/hg19 genome assembly. These primers assessed the methylation status of 6 CpG sites, 3 related to the FW and 3 related to the RV, which were included in their complementary sequence. The theoretical PCR product sizes were 110 bp for the (M) pair and 115 bp for the (U) pair due to primer length differences. PCR conditions were 5 min at 95 ºC and then 30 cycles of 20 s at 95 °C, 20 s at 55 °C and 30 s at 72 °C. DNA Gel Loading Dye (Cat#R0631, Thermo Fisher Scientific) was added to PCR products and then they were stained with ethidium bromide and visualized under UV illumination (LIAS Slite140, Avegene Life Sciences, Taipéi, Taiwan).

### In silico analysis of *BCL7A* in AML patients and cell lines

To evaluate the mutational frequency of *BCL7A* in external AML datasets, we used DepMap (data version 22Q2; disease subtype "AML", *N* = 53), TCGA-LAML (*N* = 160), and TARGET-AML (*N* = 17). TCGA/TARGET data were accessed via the Genomic Data Commons (GDC) Data Portal (Data Release 31.0). All these cohorts together account for a total of 230 patients (*N* = 230).

We downloaded *BCL7A* mRNA expression (RNA Seq V2 RSEM) and methylation (Illumina HM450 BeadChip) data of 160 AML patients from the TCGA Acute Myeloid Leukemia Project (TCGA-LAML) via cBioPortal (https://www.cbioportal.org/). Similarly, we downloaded methylation and gene expression values from the TARGET-AML cohort using the R package ‘TCGAbiolinks’, as well as from Glass et al. [15] from Gene Expression Omnibus (accession IDs: GSE6891 for gene expression, GSE98350 for methylation). In all cases, methylation values were obtained as the beta value of the probe or genomic position whose expression was most anticorrelated with *BCL7A* expression, previously restricting the analysis to the *BCL7A* locus ± 400 bp. We also obtained gene expression and reduced-representation bisulfite sequencing (RRBS-Seq) data of 32 AML cell lines from DepMap portal (https://depmap.org/portal/). Negative correlations between expression and beta values of individual CpGs were assessed using the Pearson correlation coefficient and p-values were FDR-corrected for multiple comparisons.

### Bisulfite sequencing

For bisulfite conversion, EZ DNA Methylation-Gold™ Kits (Cat#D5005, Zymo Research, Irvine, CA, USA) were used. Additionally, commercial genomic DNAs that were completely methylated (gDNA Meth) and unmethylated (gDNA Unmeth) (Cat#59,695, QIAGEN, Hilden, Germany) were used as controls.

The TA-cloning system was performed to get quantitative bisulfite sequencing results. After bisulfite conversion, the DNA was amplified. A 579 bp fragment was studied, whose genomic location was from 806 to 227 bp upstream from BCL7A translation start codon (Chr12:122,459,192—122,459,770 GRCh37/hg19 genome assembly). The primers were: BiSeq FW: 5’-TAGAAAATTTTTTAGTATTTAAGGT-3’ and BiSeq RV: 5’-TACACAAAACAAAAAAAACC-3’. This region included 61 CpG sites whose methylation status was assessed.

PCR amplification was performed using KAPA HiFi HotStart Uracil + ReadyMix PCR Kit (Cat#07,959,052,001, Roche), followed by gel purification of the PCR product using GenElute™ Gel Extraction Kit (Cat#NA1111-1KT, Merk) and A-tailing by incubation at 70 °C for 35 min using Taq DNA Polymerase (Cat#D4545, Merk), which adds an A-overhang to the insert. Then, we ligated the insert into a T-vector using pGEM®-T Easy Vector Systems (Cat#A1360, Promega, Madison, WI, USA) followed by transformation into DH5α chemically competent E. coli. and colony picking for plasmid extraction by Miniprep (Cat#21.222–4212, Biotools B & M Labs Ltd., Madrid, Spain).

Sanger sequencing (STAB VIDA Lda., Caparica, Portugal) was performed to reveal the location of methylated CpG sites by sequence alignment of the bisulfite treated DNA and the genomic DNA sequence using the pairwiseAlignment() function from the Biostrings R package (v2.52). CpG sites with sequencing errors or undetermined bases were marked as unresolved and these were not included in the analysis. Methylation fraction of a given CpG was calculated as the number of methylated reads divided by the sequencing coverage (9 inserts were sequenced, *N* = 9, Supplementary Figure 1).

### 5-aza-2'-deoxycytidine treatment

1.2 × 10^6^ NB4 cells were cultured into T-25 flasks (Cat#734-2311P, VWR) in a final volume of 6.2 mL of complete culture medium. We treated the cells with 2 µM 5-aza-2'-deoxycytidine (Decitabine, DAC) (Cat#A10292-10, Quimigen) resuspended in dimethyl sulfoxide (Cat#VWRC23486.297, VWR) for 96 h. The culture medium with DAC or only dimethyl sulfoxide (for the control condition) were renewed daily. Then, cell pellets were collected after 96 h for total RNA and total protein extraction to assess BCL7A expression levels.

### Ectopic expression of BCL7A

The plasmids HIV packaging (psPAX2) and VSV-G (pMD2.G) were acquired from Addgene (#12,260 and #12,259 respectively) and they are described elsewhere [16]. The packaging plasmid psPAX2 encodes gag, pol, tat, and rev genes, and the pMD.G plasmid encodes the vesicular stomatitis virus (VSV) G glycoprotein. The lentiviral vector (LV) pLVX-IRES-ZsGreen1 (Cat#632,187, Clontech, Shiga, Japan) features a human cytomegalovirus immediate early promoter (PCMV IE), which drives a bicistronic transcript expressing our insert and the fluorophore ZsGreen1. The pLVX-IRES-ZsGreen1 vector also contains the Woodchuck posttranscriptional regulatory element (WPRE) and the central polypurine tract (cPPT). The plasmid pUltra-Chili-Luc was acquired from Addgene (#48,688) and was used for stable expression of Luciferase in the cells used for the in vivo xenograft bioluminescence assay. The pLVX-IRES-ZsGreen1 backbone was used to clone wild-type and mutant BCL7A (Δ27-BCL7A) variants (short and long isoforms). These different inserts were generated by PCR using primers containing XbaI and BamHI restriction sites adjacent to a start codon (ATG) and a stop codon (TGA) respectively. Inserts and recipient plasmid were double digested with XbaI/BamHI and the cohesive ends ligation was performed with T4 ligase (Cat#EL0011, Thermo Fisher Scientific). The sequence of the different inserts is shown in Supplementary Figure 2.

### Lentiviral production and titration

HEK293T cells were grown in DMEM (Cat#L0103-500, Biowest, Riverside, MO, USA) supplemented with 10% fetal bovine serum, 100 U/mL streptomycin and penicillin and were plated in a 10-cm tissue culture grade Petri dish (Cat#SIAL0167, Sigma-Aldrich) the day before transfection to ensure exponential growth and 80% confluence. Vector plasmid, along with packaging (psPAX2) and envelope (pMD2.G) plasmids (18 µg total DNA; plasmid proportions of 3:2:1, respectively) were resuspended in 0.5 mL free-serum DMEM and combined at room temperature for 20 min with 45µL LipoD293 (Cat#SL100668, Signagen Laboratories, Rockville, MD, USA) previously diluted in 0.5 mL free-serum DMEM. The plasmid-lipoD293 mixture was added to the cells and incubated for 5 h. Then, it was removed and replaced by 7 mL of complete medium. The viral supernatants were collected at 48 and 72 h and filtered through a 0.45 mm filter (Cat#FPE404030, JET Biofil, Guangzhou, China), aliquoted, and immediately frozen at -80ºC.

The percentage of transduced cells was determined based on ZsGreen1 fluorescence. Viral titers (transduction units/ mL) were calculated from the percentage of ZsGreen1^+^ cells detected in the linear range of a serial dilution of the supernatant. The viral titration was estimated using K-562, a highly permissive cell line.

### Competition cell growth assay

NB4 cells were transduced with pLVX-BCL7A-IRES-ZsGreen1 coding for wild-type BCL7A, mutant BCL7A (Δ27-BCL7A), or the empty vector (EV). Cells were transduced with both short and long isoforms of wild-type and mutant BCL7A (Δ27-BCL7A) variants. We validated the expression of the different variants of BCL7A in NB4 by western blot. We transduced the cells for 3 consecutive cycles of infection at MOI = 2 with different lentiviral constructions stably co-expressing BCL7A variants along with the fluorochrome ZsGreen1. Then, transduced ZsGreen1^+^ cells were sorted and subsequently mixed with sorted non-transduced cells. The mixed cells were co-cultured and tracked by measuring the percentage of ZsGreen1^+^ population over time via Fluorescence-Activated Cell Sorting (FACS) (BD FACSVerse, BD Biosciences, San Jose, CA, USA). The percentage of ZsGreen1^+^ cells was evaluated on days 1, 5, 12, 19, and 26 and normalized versus the percentage of ZsGreen1^+^ cells at the initial time (one day after mixing).

### Xenograft in vivo bioluminescence imaging

An in vivo bioluminescence study was developed in strict compliance with the recommendations in the Guide for the Care and Use of Laboratory Animals of the Bioethical Committee of the University of Granada and the protocols were approved by the Committee on the Ethics of Animal Experiments at the University of Granada. All procedures were performed under isoflurane inhalation anesthesia, and every effort was made to minimize animal suffering.

Eight-week-old male NOD.scid Il2rgtm1Wjl/SzJ (NSG) mice (provided by the Animal Experimentation Service, University of Granada) were intravenously tail vein injected with a stable NB4 cell line (5 × 10^6^ cells/mice) that simultaneously expressed Luciferin and wild-type BCL7A/ZsGreen1 (both short and long BCL7A isoforms) or Luciferin and EV pLVX-IRES-ZsGreen1 (two groups; six mice per group). For bioluminescence imaging, mice were injected intraperitoneally with D-luciferin solution dissolved in phosphate-buffered saline (PBS) at a dose of 150 mg/kg body weight. After 5 to 8 min, the animals were anesthetized in a dark chamber using 3% isoflurane in the air at 1.5 L/min and O_2_ at 0.2 L/min/mouse, and animals were imaged in a chamber connected to a camera (IVIS, Xenogen, Alameda, CA, USA). Exposure time was 3 min in large binning, and the quantification of light emission was performed in photons/second using Living Image software (Xenogen). Tumor growth was monitored on days 0, 7, 14, and 21 by in vivo imaging and bioluminescence measurement performed in ventral and dorsal positions.

### Western blot

Total protein was extracted using RIPA lysis buffer (150 mM NaCl, 1% NP-40, 0.5% sodium deoxycholate, 0.1% SDS and 50 mM Tris–HCl pH 7.5) containing protease and phosphatase inhibitors (0.2 mM PMSF, 7 mM VO_4_ and 1 × complete Mini EDTA-free Protease Inhibitor Cocktail Tablets). Lysates were subjected to denaturing Western blot analysis to determine BLC7A (#HPA019762, Sigma-Aldrich) protein levels. Finally, β-actin (Cat#A5441, Sigma Aldrich, Merck KGaA, Darmstadt, Germany) was used as loading control. Chemiluminescence western blotting analysis system IQ LAS 4000 (GE Healthcare) was used to get the images. Band quantification was carried out by ImageJ v1.52a software. We normalized the data with β-actin values.

### Quantitative real-time PCR analysis of BCL7A

We optimized a quantitative real-time PCR assay (qRT-PCR) for the detection of *BCL7A* mRNA using SYBR Green (Cat#KK4618, Sigma-Aldrich) and specific primers: BCL7A-Fw: 5´-GTGACACATCCCTACGAATCTAC-3´ and BCL7A-Rv: 5´-CACTTCTCGTCCTTGCCTTT-3´. One microgram of RNA was used for reverse transcription with RevertAid RT Kit (Cat#K1691, Thermo Fisher Scientific) using random primers and following the manufacturer's instructions. PCR reactions contained 30 ng total cDNA, 5 µl SYBR Green 2 × and 0.2 µl of each primer (10 µM). PCR conditions were 2 min at 50 ºC, 10 min at 95 ºC, 15 s at 95 ºC and 1 min at 60 ºC for 40 cycles. All samples were measured in triplicate. For each experimental sample, the amount of *BCL7A* and endogenous reference (*GAPDH*) were determined. Expression levels of *BCL7A* and *GAPDH* were calculated by the standard curve method and the mean log_2_(*BCL7A*/*GAPDH* expression) was calculated for each sample.

### RNA sequencing

Total RNA extraction was carried out using *mir*Vana™ miRNA Isolation Kit, with phenol (Cat#AM1560, Thermo Fisher Scientific). We extracted total RNA from 3 × 10^6^ of NB4 cells of each condition (wild-type BCL7A and mutant BCL7A, described previously in the competition cell growth assay methods) per sample. Two samples per condition were prepared. RNA concentration and quality were measured using Qubit 4 Fluorometer (ThermoFisher Scientific) and 2100 Bioanalyzer Instrument (Agilent Technologies, Santa Clara, CA, USA) respectively. RNA samples were stored at -80ºC until they were shipped to Genewiz (a division of Azenta Life Sciences), where RNA-seq was performed (Illumina NovaSeq, 2 × 150 bp configuration).

Quality control of the raw FASTQ files was performed using FastQC (v0.11.8). Reads were aligned to the human genome GRCh38.d1.vd1 (downloaded from https://gdc.cancer.gov/about-data/data-harmonization-and-generation/gdc-reference-files) using STAR (version 2.7.10b) in “two-pass mode”. GENCODE v36 was used as an annotation reference. The multi-sample BAM was split into single-sample BAMs. Quality of the BAM files was assessed using Picard (v1.4.2) and QualiMap (v2.2.1) in combination with multiqc (v1.6). We checked that rRNA contamination was < 1% and we used Integrative Genomics Viewer to confirm the presence of the expected BCL7A mutations at the RNA level.

Read counts per gene were obtained using htseq-count (v0.6.1p1) with the following options: “-m intersection-nonempty -f bam -t exon -i gene_id -r pos -s reverse”. Further analyses were performed using R (version 3.6.3, Bioconductor version 3.10) and the packages ‘edgeR’, ‘DESeq2’ and ‘EnhancedVolcano’. Hierarchical clustering and principal component analysis were performed to confirm the similarities among replicates and conditions. DESeq2 was used for the differential expression analysis testing for abs(logFC) > log_2_(1.5) specifying a threshold of alpha = 0.01. logFC values were shrunk using the ashr method while preserving the original p and adjusted p values. Gene ontology (GO) enrichment analysis was carried out using the gene annotation and analysis resource Metascape (http://metascape.org/gp/index.html) [17] using the top 400 differently expressed genes according to their *p*-adjusted value.

### Statistical analyses

Unless specified otherwise, all statistical analyses were performed using R (version 3.6.1). Normality of continuous data was assessed using Q-Q plots and Shapiro–Wilk tests and the subsequent statistical tests were chosen accordingly.

## Results

### BCL7A expression is modulated in AML patients by promoter methylation

**Fig. 1:**
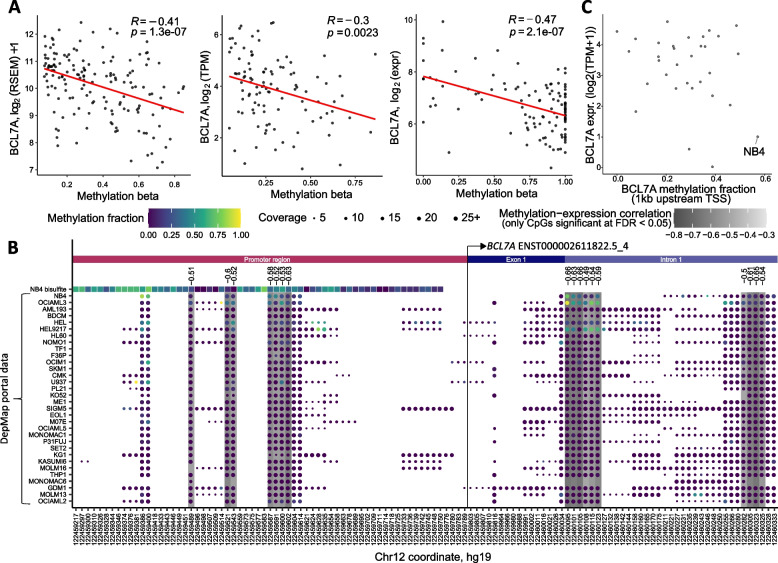
*BCL7A* methylation and expression status in AML patients and cell lines. **a** Significant inverse correlation (Pearson’s correlation coefficient) between methylation and expression of BCL7A in TCGA-LAML cohort, TARGET-AML and Glass et al. Methylation is expressed as the beta-value of the Illumina HM450 probe (TCGA, TARGET) or genomic position (Glass et al.) most inversely correlated with gene expression. **b** CpG methylation profile around the transcription start site (TSS) of *BCL7A* in AML cell lines. Methylation fraction measured by reduced representation bisulfite sequencing data from DepMap Portal (https://depmap.org/portal/) is shown along with our bisulfite sequencing data of the NB4 cell line (top row). CpGs significantly correlated with expression (FDR < 0.05) are highlighted under grey background and their Pearson’s correlation coefficient is shown above. The TSS of *BCL7A* is indicated by a vertical line. **c** Methylation fraction 1 kb upstream of the *BCL7A* TSS and gene expression in 32 AML cell lines from DepMap Portal. NB4 shows the highest methylation level as well as low BCL7A expression

To study the involvement of *BCL7A* in AML, first, we checked its mutation frequency in public AML datasets. Consistent with the low mutational burden of AML, we found a very low frequency of *BCL7A* mutations in AML samples (< 1% non-synonymous coding mutations; *N* = 230; see Methods), ruling out mutations as a mechanism of *BCL7A* inactivation in AML. Then, we examined possible mechanisms of epigenetic expression inactivation by assessing *BCL7A* methylation levels in three independent AML cohorts: TCGA-LAML (*N* = 160), TARGET-AML (*N* = 188), and Glass et al. [15] (*N* = 111). We observed a significant negative correlation between *BCL7A* promoter methylation and *BCL7A* mRNA expression in three independent AML patient cohorts (Fig. 1a), suggesting that differential methylation of the *BCL7A* promoter may affect *BCL7A* mRNA expression in AML.

BCL7A expression is silenced in an AML cell line model due to promoter hypermethylation.

**Fig. 2:**
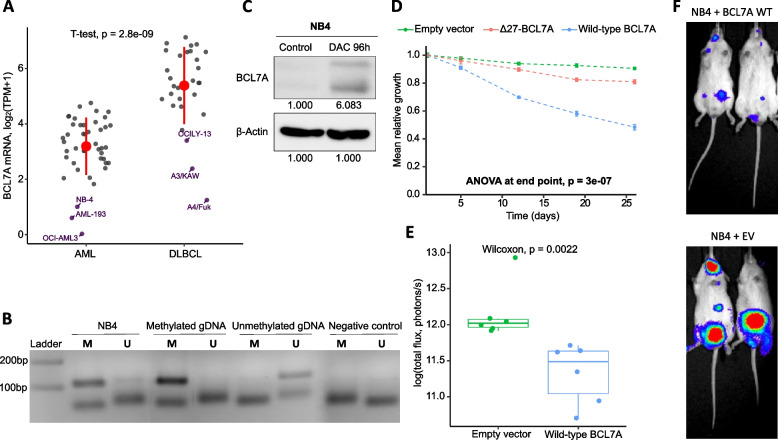
*BCL7A* restoration promotes a tumor suppressor-like phenotype. **a** Differential expression of *BCL7A* mRNA between acute myeloid leukemia (AML) and diffuse large B-cell lymphoma (DLBCL) cell lines from DepMap. TPM: transcripts per million (Supplementary Table 1). **b** Methylation Specific PCR (MSP). PCR products generated by the specific primers for the methylated sequence (M) and for the unmethylated sequence (U) are depicted. MSP was carried out using genomic DNA from the NB4 cell line, as well as completely methylated and unmethylated genomic DNAs. In addition, a negative control with no DNA template was included. **c** Relative BCL7A protein expression differences of the NB4 cell line due to Decitabine (DAC) treatment. BCL7A presents two alternative isoforms that are co-expressed due to an extended exon variant located in exon 5, and due to this, there are two bands for BCL7A. β-actin was used as a loading control and the numeric result was normalized with this loading control signal. **d** Competition cell growth effect of BCL7A expression restoration on in vitro proliferation. Competition cell growth assay of non-transduced NB4 cells vs NB4 cells transduced with either pLVX-IRES-ZsGreen1 plasmid expressing wild-type BCL7A, mutant BCL7A (∆27-BCL7A), or empty vector. All constructs were evaluated in a time course of ZsGreen1.^+^ % measurements on days 1, 5, 12, 19, and 26. This assay involves the co-culture of an NB4 model transduced with one of the plasmids together with non-transduced NB4 cells. **e** Bioluminescence signal at day 21 after injection of NB4-luciferase-transduced cells in the empty vector mice group (*n* = 6) and wild-type BCL7A mice group (*n* = 6) groups. **f** Image of the bioluminescence signal of two representative NGS mice in ventral position on day 21 after the tail-injection of NB4-luciferase cells transduced with BCL7A wildtype (up) and empty vector (down)

In order to establish a functional cell line model to study the role of *BCL7A* in AML, we analyzed data from the DepMap database and found that the NB4 cell line had the highest promoter methylation level as well as low *BCL7A* expression (Fig. 1b, c). Also, we reanalyzed *BCL7A* mRNA expression data from DepMap (22Q2) and tested the differential expression in AML compared to DLBCL cell lines, since it is well known the implication of *BCL7A* in DLBCL. AML cell lines consistently had lower *BCL7A* expression than DLBCL cell lines (fold change = -4.6, p = 2.8 × 10^–9^, Fig. 2a and Supplementary Table 1). Interestingly, among the myeloid cell lines, NB4 was found among the AML cell lines with the lowest expression level of *BCL7A* mRNA (Fig. 2a, Supplementary Table 1). Therefore, the NB4 cell line was selected to further analyze the epigenetic mechanisms leading to *BCL7A* inactivation.

To rule out that low *BCL7A* expression in NB4 cell line is due to a genetic alteration, *BCL7A* coding sequence was sequenced at the genomic DNA level using previously published protocols and primers [3]. No mutations were found in its coding sequence. Then, we studied the methylation status of the *BCL7A* promoter in the NB4 cell line by two independent approaches. First, we followed a previously described methylation-specific PCR (MSP) protocol [14] (see Methods section). Our MSP results showed that the *BCL7A* promoter was hypermethylated in NB4 cells (Fig. 2b). Second, we performed bisulfite sequencing across a DNA region within the *BCL7A* proximal promoter comprising 61 CpG sites. A total of 33.52% of the captured CpG sites were methylated (upper row of Fig. 1b and Supplementary Figure 1). In accordance with DepMap, our data showed that the *BCL7A* promoter is hypermethylated in the NB4 cell line. Whereas our bisulfite sequencing experiment covered each CpG along the proximal promoter region, DepMap coverage was incomplete. Regardless, our experimental results closely matched those of DepMap for the NB4 cell line in all CpGs covered by DepMap.

Taking advantage of methylation and expression data collected in DepMap, we could also determine that the methylation status of 17 CpGs had a significant negative correlation with gene expression (Fig. 1b). Some of these CpGs were located in the proximal promoter while others were located within the first intron, the latter being a phenomenon that has been negatively correlated with gene expression [18]. To further test the relationship between promoter methylation and *BCL7A* expression, we checked if *BCL7A* expression could be restored by using a demethylating agent such as 5-aza-2’-deoxycytidine (Decitabine, DAC). Indeed, BCL7A protein expression was rescued after DAC treatment (Fig. 2c and Supplementary Figure 3). Overall, these results confirmed that *BCL7A* silencing in NB4 is due to promoter hypermethylation and that its expression could be pharmacologically restored in vitro.

### BCL7A presents a tumor suppressor role in an AML cell model both in vitro and in vivo

Next, we wondered whether BCL7A expression restoration could restrain the tumoral phenotype in the NB4 cell line. To assess this, we performed a growth competition assay upon BCL7A expression restoration. For this purpose, we transduced the NB4 cell line with lentiviral constructs co-expressing the fluorochrome ZsGreen1 along with different BCL7A variants: i) wild-type BCL7A (WT) ii) mutant BCL7A (∆27-BCL7A) [3]; and iii) an empty vector (EV) (Supplementary Figure 2). Mutant BCL7A (∆27-BCL7A) was used as a control since it lacks 27 amino acids that are necessary for it to bind to SMARCA4 and assemble to the SWI/SNF complex [3, 6, 19] (Supplementary Figure 4). Cells transduced with any of the three constructs were co-cultured with non-transduced NB4 cells. The NB4 cell line expressing wild-type BCL7A had reduced growth when co-cultured with non-transduced NB4 cells in comparison with the NB4 cell line expressing either mutant BCL7A (∆27-BCL7A) or the EV (Fig. 2d). These results support that BCL7A plays a tumor suppressor role in the NB4 myeloid cell line.

To extend these results to an additional cell line from another AML subtype we used M-07e, which has average levels of *BCL7A* expression and *BCL7A* promoter methylation according to the AML DepMap collection (Supplementary Figure 5). Competition cell growth assay developed in the M-07e cell line displayed similar results to NB4, in which the expression of wild-type BCL7A in the M-07e cell line decreased its fitness significantly more than the empty vector and mutant BCL7A (Δ27-BCL7A) (Supplementary Figure 6).

Furthermore, we evaluated the tumor suppressor activity of BCL7A through in vivo xenograft studies. For this purpose, first, we generated NB4 cells stably expressing luciferase (see methods section). Next, we transduced the luciferase-expressing NB4 cells with either wild-type (WT) BCL7A or the empty vector (EV) (Supplementary Figure 2). Then, we intravenously injected five million cells per mouse transduced with either WT or EV in two groups of six NSG immunodeficient mice. We applied in vivo image technologies (IVIS-Spectrum) to monitor the bioluminescence. After 21 days, mice injected with BCL7A WT-expressing cells showed significantly lower bioluminescence compared to mice injected with control cells (Fig. 2e, f). The bioluminescence reduction evinced a lower tumorigenic ability in the presence of BCL7A, corroborating its tumor suppressor role in vivo. Therefore, our in vivo experiments confirmed that BCL7A expression restoration promotes a tumor suppressor phenotype in NB4 cells.

### *BCL7A* expression restoration alters the expression of key genes of AML

**Fig. 3:**
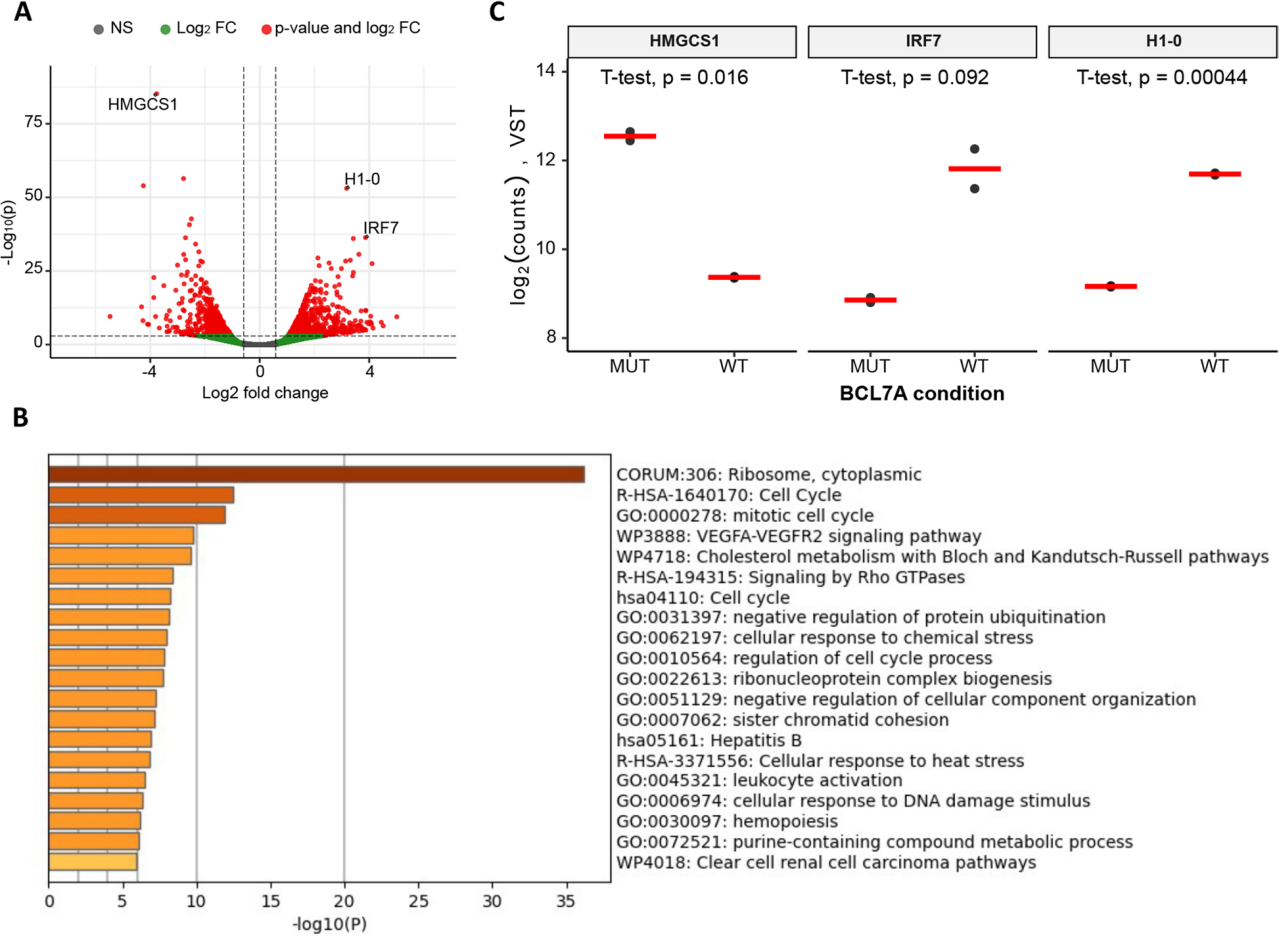
RNA sequencing of wild-type BCL7A vs mutant BCL7A NB4 cell models. **a **Volcano plots highlighting the differentially expressed genes in NB4 cell line when comparing NB4 transfected with BCL7A wildtype and BCL7A mutant. **b** Enriched terms across the top significant (p adjusted) 400 differentially expressed genes when comparing NB4 transduced with wild-type BCL7A and BCL7A mutant. **c** Expression values according to our RNA-seq data of three selected genes: HMGCS1, IRF7, and H1-0. Log_2_ of variance-stabilized transformed (VST) gene counts was obtained with DESeq2 in R. The red horizontal lines represent the mean

To gain more insight into the functional consequences of restoring BCL7A expression, we performed RNA sequencing in the NB4 cell line, comparing wild-type BCL7A and mutant BCL7A transcriptomes. We identified 1151 differentially expressed genes (adjusted *p*-value < 0.001; Fig. 3a and Supplementary Table 2). To determine if the altered genes cluster in any functional pathway, we performed a GO enrichment analysis using the top 400 genes with better-adjusted p-value and the algorithm Metascape [17]. The top significant enriched pathways (*p* < 1 × 10^–10^) were related to protein synthesis, cell cycle, and mitotic cell cycle (Fig. 3b), which fits with the phenotype observed during the NB4 competition cell growth assay.

In the comparison NB4 transduced cells with wild-type BCL7A vs mutant BCL7A (Δ27-BCL7A), the most differentially negatively expressed gene according to its adjusted p-value was *HMGCS1* (p-adjusted value = 9.12 × 10^–82^, 13.63 fold change of downregulation; Fig. 3c). Importantly, *HMGCS1* is a key enzyme in the mevalonate pathway of cholesterol synthesis with oncogenic activities in AML patients where has been found overexpressed in newly diagnosed and relapsed/refractory patients [20]. The most differentially positively expressed genes according to their adjusted *p*-value were *H1-0* (p-adjusted value = 8.36 × 10^–54^, 9.02 fold change) and *IRF7* (p-adjusted value = 8.75 × 10^–34^, 14.58 fold change). The observed changes in *H1-0* expression could be related to the expected chromatin activity, since *H1-0* codifies a linker histone that is involved in the formation of supra-nucleosomal chromatin higher-order structures. Additionally, *H1-0* is an epigenetic regulator of cell proliferation and differentiation [21]. On the other hand, Interferon regulatory factor 7 (*IRF7*) has been found to have tumor suppressor properties in AML since the *IRF7* loss accelerates acute myeloid leukemia progression while *IRF7* overexpression in decreased leukemia stem cell (LSC) levels [22].

Taken together, our results suggest that *BCL7A* modulates the expression of key genes that can be linked to a tumor suppressor phenotype in the AML context.

## Discussion

Over the last decade, sequencing studies have shown that the SWI/SNF complex is among the most frequently mutated functional elements in cancer [7]. Most of these mutations are deleterious and suggest a tumor suppressor activity for this complex. In AML, however, functional studies have challenged this tumor suppressor activity, or at least suggested that the SWI/SNF complex may be necessary to support leukemogenesis [6]. In this regard, the SWI/SNF catalytic subunit SMARCA4 was reported to play a role in AML maintenance, probably by regulating MYC expression [11, 12]. Similarly, BRD9 was found to be required in AML cells to sustain MYC transcription, rapid cell proliferation, and differentiation blockage [23]. Moreover, BCL11A has been found to promote myeloid leukemogenesis by repressing PU.1 target genes [24]. However, recent functional studies have shown that SWI/SNF complexes may exhibit a tumor suppressor role in some contexts [25]. For example, in mice, Arid2 has a dual role, being suppressed to enhance initial leukemogenesis, but being necessary during leukemia maintenance [25]. On the other hand, in the same mouse models, suppression of Arid1b at either stage promoted leukemogenesis. In another study, where *ARID1A* and *ARID1B* were found frequently harboring loss of function mutations in acute promyelocytic leukemia, ARID1B deficiency caused a block in differentiation in this leukemia subtype [9]. Moreover, sequencing studies carried out on AML and/or myelodysplastic syndromes (MDS) have found recurrent deleterious mutations in SWI/SNF genes including *ARID2* [26], *ARID1A* and *ARID1B* [9], *SMARCA2* [27], and *SMARCA4* [10].

In this work, we have shown that expression of the SWI/SNF subunit *BCL7A* is inversely correlated to genomic DNA methylation in three independent cohorts of AML patients. In addition, we found an AML cell line model (NB4) where BCL7A expression is inactivated due to promoter hypermethylation, and that this inactivation is reversible using DNA methyltransferase inhibitors. Restoration of BCL7A expression in cell culture impaired cell proliferation in competition cell growth assay and tumor formation on xenografts in vivo, altering the expression of genes like *HMGCS1, H1-0, IRF7*, which may help to explain its tumor suppressor role in AML [20–22]. Interestingly, from a clinical perspective, these observations may explain, at least in part, the effectiveness of DNA methyltransferase inhibitors currently approved for clinical use in patients with myeloid neoplasms [28]. Currently, the activity of the SWI/SNF complex has gained increasing clinical interest for targeted therapies due to the discovery of several synthetic lethal interactions between subunits [29–31]. Significantly, these therapies have been explored in AML, and a recent report supported the idea of targeting SWI/SNF catalytic function as a potential therapeutic strategy in AML [32]. Additionally, drugs that target the BRD9 subunit have an anti-tumoral phenotype in AML [33]. Although these results are suggestive, further research will help determine the extent of SWI/SNF complex on AML and its potential value in the clinic.

## Conclusions

In this study, we show for the first time that expression of the SWI/SNF complex subunit *BCL7A* was inversely correlated with promoter methylation in AML patients. In addition, we have found an AML cell line (NB4) in which *BCL7A* expression is inactivated due to promoter hypermethylation. Restoration of BCL7A hindered cell proliferation in competition cell growth assay and xenograft tumor formation in vivo modulating significantly the expression of genes such as *HMGCS1, H1-0, and IRF7*, which may help to explain its tumor suppressor role in AML. These findings highlight the importance of *BCL7A* in hematological malignancies, expanding its role not only in lymphoma but also in the pathogenesis of myeloid leukemias.

## Supplementary Information

**Additional file 1:** **Supplementary Figure 1. **Diagram displaying CpG-methylation status around the *BCL7A *TSS. Genomic DNA from the NB4 cell line was subjected to bisulfite conversion and used for subsequent TA-cloning. **Supplementary Figure 2. **Schematic representation of the different lentiviral plasmids used in the experimental procedures. The specific region of the long isoform of BCL7A is colored in blue. **Supplementary Figure 3. **Western blot including the Decitabine (DAC) treatment over the NB4 cell line shown in Fig. 2c. **Supplementary Figure 4. **Protein-protein interactions between BCL7A and SMARCA4 as determined by Mashtalir et al (2020). **Supplementary Figure 5. **DepMap AML cell lines collection data showing BCL7A Methylation Fraction (1kb upstream TSS) vs BCL7Aexpression level. NB4 and M07e are marked. **Supplementary Figure 6. **Competition cell growth effect of BCL7A expression restoration *on in vitro* proliferation. **Supplementary Table 1.**

**Additional file 2:** **Supplementary Table 2.** Differential expression analysis results.

## Data Availability

RNA-seq data discussed in this publication is accessible through Gene Expression Omnibus under accession number GSE226604.

## Abbreviations

AML: Acute myeloid leukemia
APL: Acute promyelocytic leukemia
DAC: 5-Aza-2’-deoxycytidine (Decitabine)
DLBCL: Diffuse large B-cell lymphoma
EV: Empty vector
MSP: Methylation-specific PCR
SWI/SNF: Switch/Sugar Non-Fermenting
∆27-BCL7A: Mutant BCL7A
