# Supplementary material for: BCL7A is silenced by hypermethylation to promote acute myeloid leukemia
Source: Biomark Res. 2023 Mar 20;11:32. doi: 10.1186/s40364-023-00472-x (PMC10026484; doi:10.1186/s40364-023-00472-x)
Supplement: Supplementary file 1 — Additional file 1: Supplementary Figure 1. Diagram displaying CpG-methylation status around the BCL7A TSS. Genomic DNA from the NB4 cell line was subjected to bisulfite conversion and used for subsequent TA-cloning. Supplementary Figure 2. Schematic representation of the different lentiviral plasmids used in the experimental procedures. The specific region of the long isoform of BCL7A is colored in blue. Supplementary Figure 3. Western blot including the Decitabine (DAC) treatment over the NB4 cell line shown in Fig. 2c. Supplementary Figure 4. Protein-protein interactions between BCL7A and SMARCA4 as determined by Mashtalir et al (2020). Supplementary Figure 5. DepMap AML cell lines collection data showing BCL7A Methylation Fraction (1kb upstream TSS) vs BCL7Aexpression level. NB4 and M07e are marked. Supplementary Figure 6. Competition cell growth effect of BCL7A expression restoration on in vitro proliferation. Supplementary Table 1. [file 40364_2023_472_MOESM1_ESM.pdf]

Supplementary Figure 1

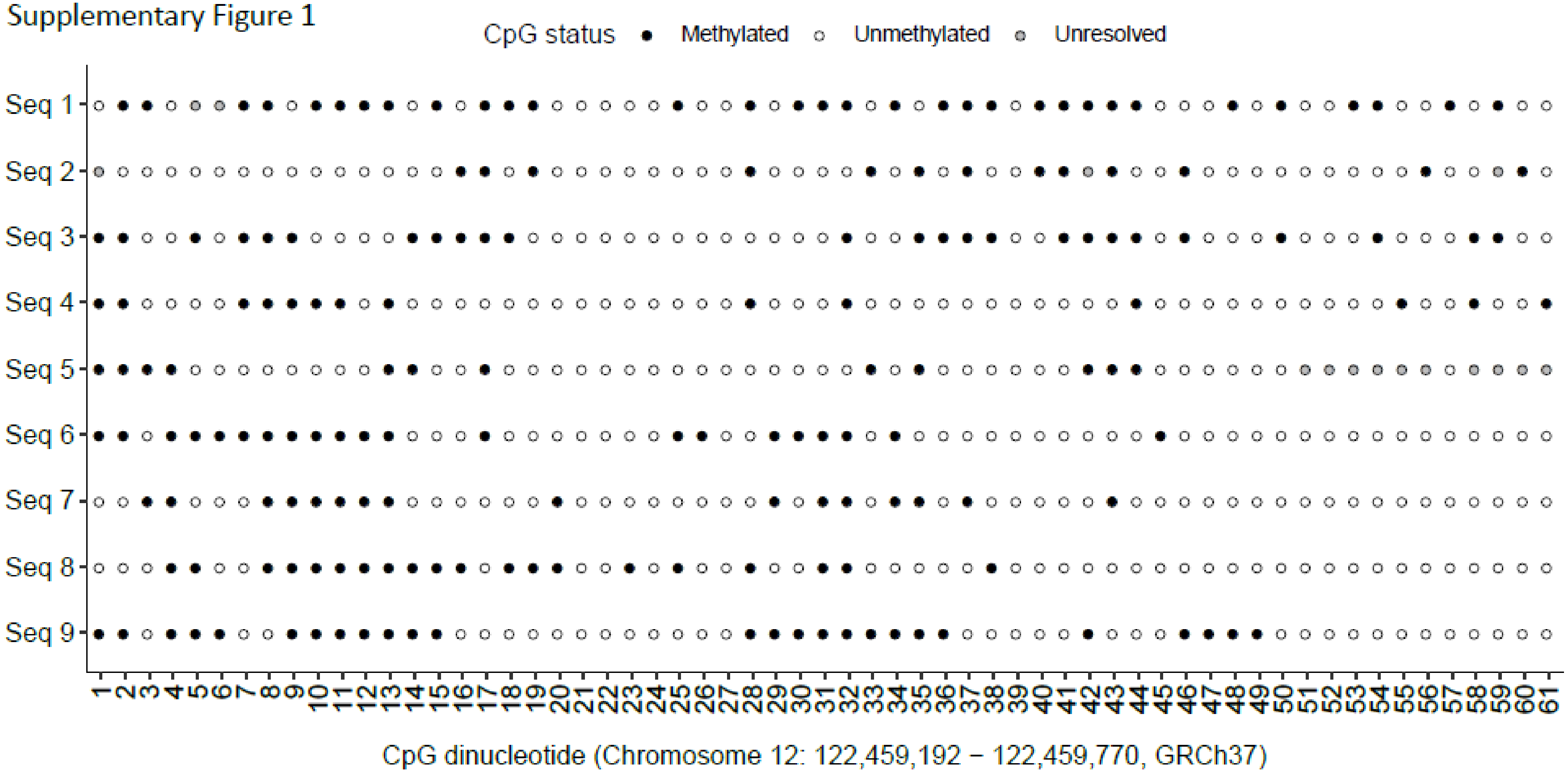

Supplementary Figure 1. Diagram displaying CpG-methylation status around the *BCL7A* TSS. Genomic DNA from the NB4 cell line was subjected to bisulfite conversion and used for subsequent TA-cloning. Then, nine independent amplicons were analysed by Sanger sequencing. 61 CpG sites included in the 579 bp amplicon (chr12:122,459,192 - 122,459,770 GRCh37; hg19 genome assembly) were assessed.

Supplementary Figure 2

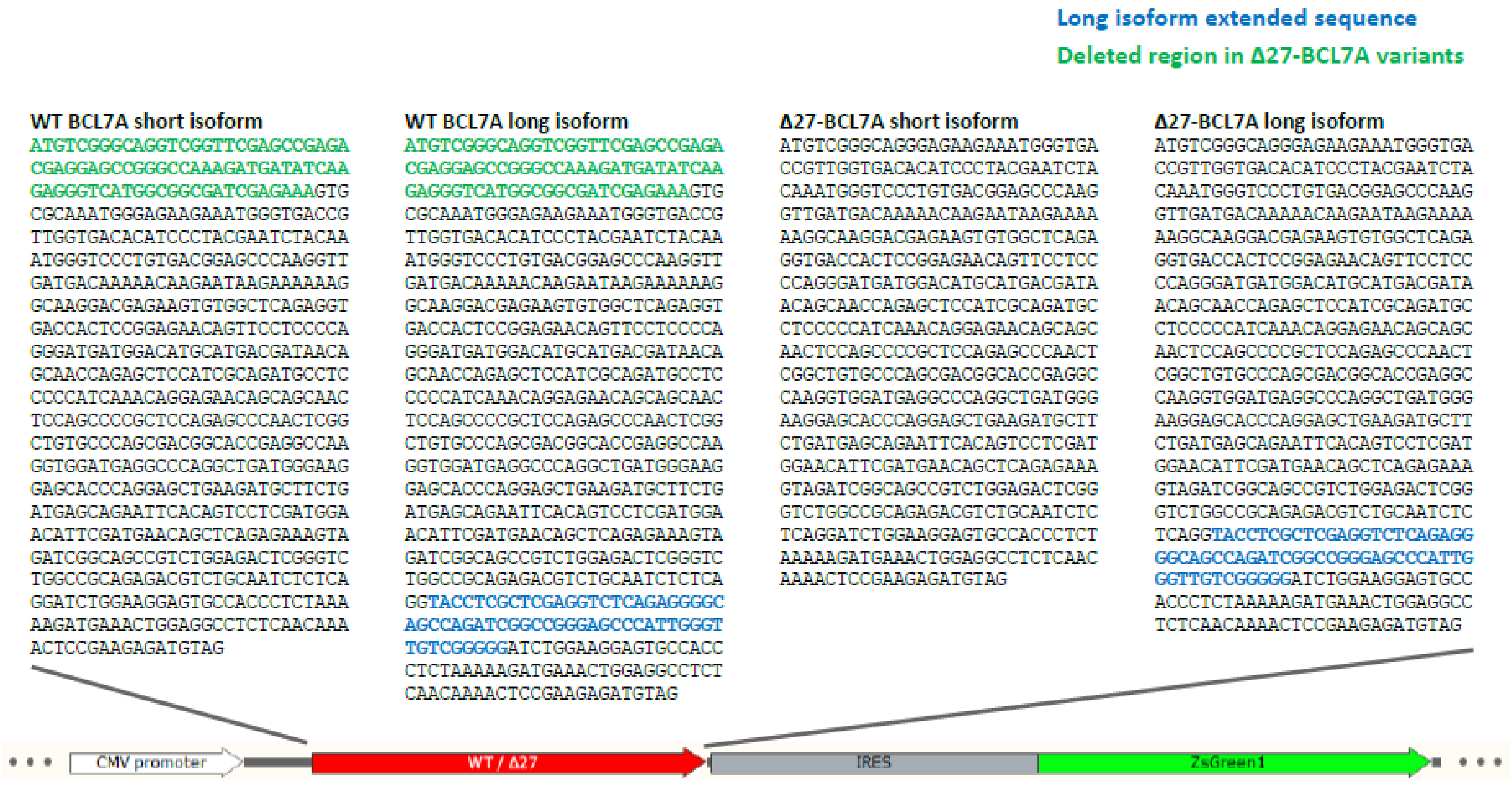

Lentiviral plasmid expressing *BCL7A* versions

Supplementary Figure 2. Schematic representation of the different lentiviral plasmids used in the experimental procedures. The specific region of the long isoform of BCL7A is colored in blue. The specific region that is deleted in the BCL7A mutant (Δ27-BCL7A) is colored in green. The Empty Vector (EV) has the pLVX-IRES-ZsGreen1 structure without any insert.

Supplementary Figure 3

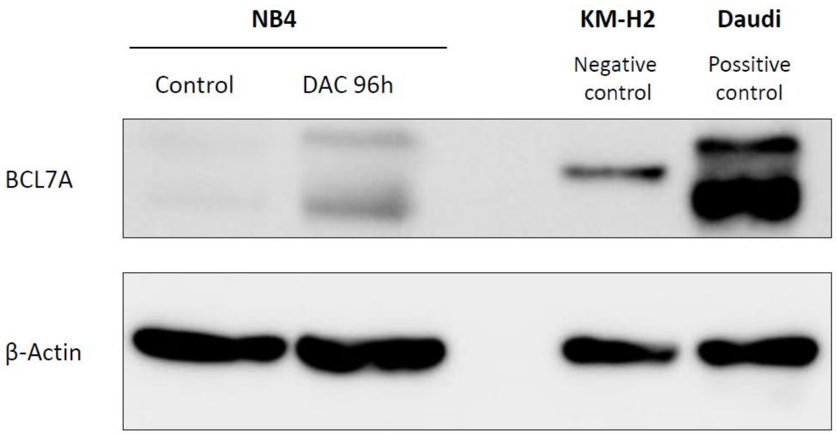

Supplementary Figure 3. Western blot including the Decitabine (DAC) treatment over the NB4 cell line shown in Fig. 2c. Here we also show the rest of the lanes including a negative control of BCL7A expression (KM-H2 cell line, which has no BCL7A expression), and a positive control (Daudi cell line) which expresses average BCL7A levels. The band which appears in KM-H2 sample is an inespecific band due to an inespecific binding of the anti-BCL7A (Cat#HPA019762) that we used.

Supplementary Figure 4

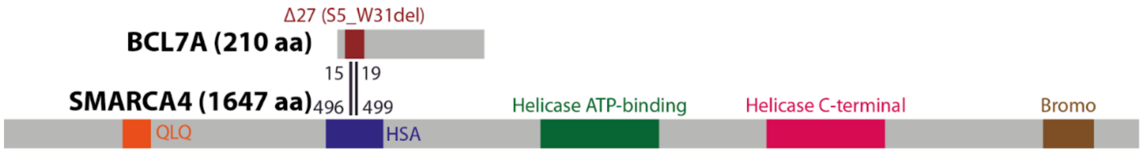

Supplementary Figure 4. Protein-protein interactions between BCL7A and SMARCA4 as determined by Mashtalir et al (2020). The numbers indicate the amino acid positions that interact with each other (solid lines). The region from exon 1 that is deleted in splice site mutant BCL7A (Δ27) is marked in red. Known domains of SMARCA4 as described in UniProt are also marked. HSA: helicase/SANT-associated.

Supplementary Figure 5

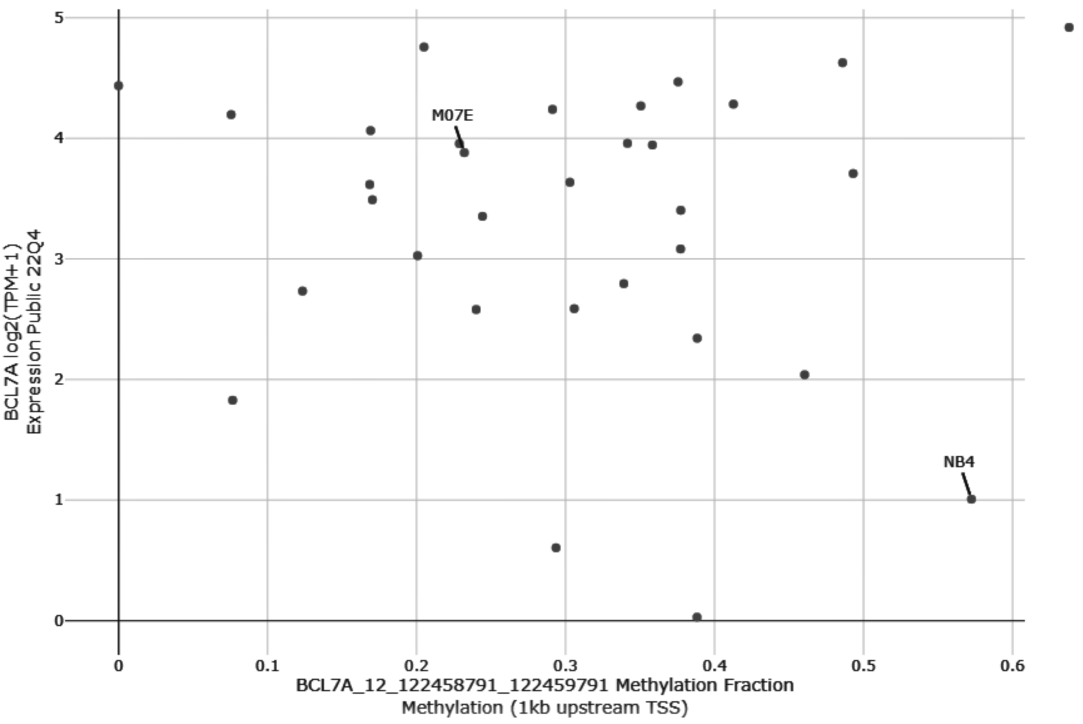

Supplementary Figure 5. DepMap AML cell lines collection data showing BCL7A Methylation Fraction (1kb upstream TSS) vs BCL7A expression level. NB4 and M07e are marked.

Supplementary Figure 6

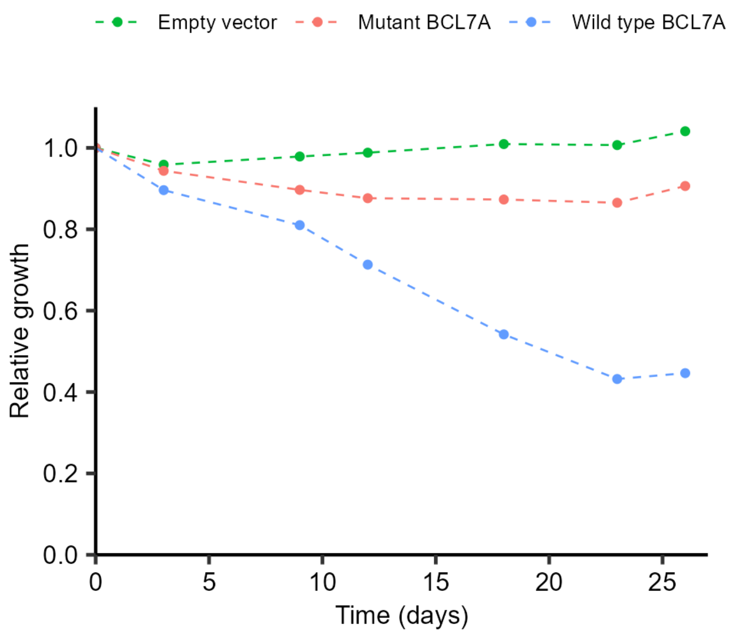

Supplementary Figure 6. Competition cell growth effect of BCL7A expression restoration *on in vitro* proliferation. Competition cell growth assay of non-transduced M-07e cells vs M-07e cells transduced with either pLVX-IRES-ZsGreen1 plasmid expressing wild-type BCL7A, mutant BCL7A (Δ27-BCL7A), or empty vector. All constructs were evaluated in a time course of ZsGreen1+ % measurements on days 0, 3, 9, 12, 18, 23 and 26. This assay involves the co-culture of an M-07e model transduced with one of the plasmids together with non-transduced M-07e cells.

Supplementary Table 1

Cell line	Subtype	BCL7A mRNA, log2(TPM+1)
OCI-AML3	AML	0,028569152
AML-193	AML	0,604071324
NB4	AML	1,007195501
BDCM	AML	1,827819025
HEL	AML	2,039138394
OCI-M2	AML	2,056583528
HEL 92.1.7	AML	2,341985747
NA	AML	2,40053793
HL-60	AML	2,580145484
MUTZ-8	AML	2,587364991
NOMO-1	AML	2,587364991
TF-1	AML	2,733354341
F-36P	AML	2,752748591
OCI-M1	AML	2,794935663
MOLM-14	AML	2,944858446
SHI-1	AML	3,016139703
OCI-AML4	AML	3,025028794
SKM-1	AML	3,026800059
CMK	AML	3,08236197
NA	AML	3,116031993
CMK-11-5	AML	3,122672719
SKNO-1	AML	3,148934105
MV4;11	AML	3,277984747
U-937	AML	3,353323291
PL-21	AML	3,49057013
KO52	AML	3,614709844
KASUMI-1	AML	3,626439137
ME-1	AML	3,635754391
SIG-M5	AML	3,708187236
EOL-1	AML	3,786596362
M-07e	AML	3,881664619
OCI-AML5	AML	3,944858446
MONO-MAC-1	AML	3,956056652
P31/FUJ	AML	3,957914599
NA	AML	3,9800253
Set-2	AML	4,06436554
KG-1	AML	4,196134881
Kasumi-6	AML	4,239550797
MOLM-16	AML	4,268284667
THP-1	AML	4,283180979
MONO-MAC-6	AML	4,436961338
GDM-1	AML	4,467931546
MOLM-13	AML	4,627023106
OCI-AML2	AML	4,757023247

Cell line	Subtype	BCL7A mRNA, log2(TPM+1)
A4/Fuk	DLBCL	1,250961574
A3/KAW	DLBCL	2,386810946
OCILY-13	DLBCL	3,399171094
KML-1	DLBCL	3,777156666
SU-DHL-8	DLBCL	4,341274184
RC-K8	DLBCL	4,622930351
HT	DLBCL	4,657068301
NU-DHL-1	DLBCL	4,894817763
OCI-LY3	DLBCL	4,905447179
WSU-NHL	DLBCL	5,058316496
RL	DLBCL	5,086189313
SU-DHL-5	DLBCL	5,298291731
OCI-LY-19	DLBCL	5,362469889
OCI-LY7	DLBCL	5,479618608
Farage	DLBCL	5,482202926
OCI-LY18	DLBCL	5,560714954
NU-DUL-1	DLBCL	5,646738698
SU-DHL-4	DLBCL	5,935223994
Pfeiffer	DLBCL	6,013238915
SU-DHL-10	DLBCL	6,31596467
DB	DLBCL	6,464831606
WSU-DLCL2	DLBCL	6,594399256
Toledo	DLBCL	6,599020177
VAL	DLBCL	6,627606838
SU-DHL-6	DLBCL	6,657354182
DOHH-2	DLBCL	6,827692021
U-2904	DLBCL	6,874059203
KARPAS-422	DLBCL	6,912649865
RI-1	DLBCL	7,126601235

Supplementary Table 1. *BCL7A* mRNA expression level in log2(TPM+1) and subtype of the cell lines included in Fig. 2a. Data from DepMap Portal database (22Q2).
